# Parental imprinting effects on milk yield and composition traits in Murciano-Granadina goats

**DOI:** 10.1016/j.vas.2026.100915

**Published:** 2026-10-26

**Authors:** Morteza Mokhtari, Farhad Ghafouri-Kesbi, Moslem Moghbeli Damane

**Affiliations:** a Department of Animal Science, Faculty of Agriculture, University of Jiroft, P.O. Box 364, Jiroft, Iran; b Department of Animal Sciences, Faculty of Agriculture, Bu-Ali Sina University, Hamedan, Iran

**Keywords:** Animal model, Imprinting effects, Milk yield and content, Variance components, Goat

## Abstract

Parental imprinting, a parent-of-origin effect usually ignored in genetic evaluations, could influence milk yield and composition traits as well as the accuracy of breeding values. This study therefore examined its impact on these traits over the first three lactations in Murciano-Granadina goats. The dataset consisted of pedigree and test-day records for milk yield and composition traits collected during the first three lactations of Murciano-Granadina goats between 2017 and 2024 at a private dairy farm in Ghale-Ganj, south of Kerman Province, Iran. The traits analyzed in the i^th^ lactation period (i = 1–3) were total milk yield (MYi), average fat percentage (FPi), average protein percentage (PPi), and average somatic cell score (SCSi), using a database consisting of 1033 to 6696 records, depending on the trait. We compared four animal models, including or excluding imprinting (maternal and paternal) effects, using Akaike’s information criterion (AIC) and average model accuracy. Model comparisons showed that parental imprinting effects were important across MY1, MY2, FP1, and FP2. Specifically, a model with direct additive and paternal imprinting effects best fit MY1 and FP1, whereas a model incorporating direct additive, paternal, and maternal imprinting best fit MY2. For these traits, the models including imprinting effects produced higher accuracies of predicted breeding values compared with models excluding imprinting effects, increasing from 3% for MY1 to 6% for MY2. Paternal imprinting effects accounted for 2% of the phenotypic variance for MY2 to 10% of the phenotypic variance for FP2. Under the best-fitting models, direct heritability ranged from low to moderate estimates, from 0.05 for FP2 to 0.34 for PP2. Genetic correlations were generally stronger than phenotypic, residual, and imprinting correlations, with milk-yield traits showing the highest genetic correlations. These findings highlight the relevance of parental imprinting in genetic evaluations of MY1, MY2, FP1, and FP2 traits and underscore the importance of model choice based on AIC and average accuracy of models for accurate selection decisions in the studied population of the Murciano-Granadina goat breed.

## Highlights

• Four animal models, differing in the extent of parental (maternal and paternal) imprinting effects, were fitted to total milk yield (MY), average fat percentage (FP), average protein percentage (PP), and average somatic cell score (SCS) in the first three lactation periods of Murciano-Granadina goats.
• Parental imprinting effects was important for MY1, MY2, FP1, and FP2 and accounted for 2% of the phenotypic variance for MY2 to 10% of the phenotypic variance for FP2.
• Under the best‑fitting models, direct heritability ranged from low to moderate estimates, from 0.05 for FP2 to 0.34 for PP2.
• Spearman’s rank correlations between models with and without imprinting were high overall but declined when rankings focused on top‑performing animals, particularly the top 1%.
• Genetic correlations were stronger than phenotypiccorrelations.

## 1 Introduction

Introducing exotic breeds to enhance key traits in local goat populations, such as increasing milk yield, can substantially improve the production efficiency of breeding systems in developing countries (Tesema & Kebede, 2022). As emphasized by Kosgey and Okeyo (2007), the livelihoods of smallholder farmers in these regions rely heavily on improving efficiency within low‑input production systems. Achieving this requires integrated improvement strategies, where enhanced management practices are complemented by well‑structured genetic improvement programs. The primary goal of any breeding program is to identify genetically superior animals, select them, and mate them to maximize genetic gain and enhance performance in subsequent generations. Developing such programs depends on obtaining accurate estimates of genetic parameters and breeding values for economically important traits.

Recently published papers have highlighted the value of incorporating parental imprinting effects into models that estimate variance components and genetic parameters for economically important traits in livestock. Evidence for this has been reported in sheep (Amiri Roudbar et al., 2017, 2018; Ghafouri-Kesbi et al., 2022; Mokhtari et al., 2022a), goats (Bahreini Behzadi, 2026; Ehsaninia & Ghafouri-Kesbi, 2024; Mokhtari et al., 2022b), and cattle (Ghafouri-Kesbi et al., 2025). Parental imprinting refers to the phenomenon in which gene expression depends on whether the allele is inherited from the mother or the father. In maternally imprinted genes, the maternal allele is partially or completely silenced in the offspring, whereas in paternally imprinted genes, the paternal allele is partially or fully silenced (Meyer & Tier, 2012).

Hill and Keightley (1988) showed that imprinting modifies the expected covariances among relatives. Within a mixed‑model framework, these effects can be accounted for by fitting models that include gametic effects (Meyer & Tier, 2012). The computational algorithm developed by Tier and Meyer (2012) for constructing and using the gametic relationship matrix made it feasible to incorporate parental imprinting effects into methods used for estimating variance components and genetic parameters.

The Murciano‑Granadina goat is a Spanish local breed recognized for its high milk yield and quality, averaging 586 kg per lactation with 5.1% fat and 3.6% protein, and for its strong adaptability to harsh environments, making it an attractive option for improving milk production in dairy goat systems (Luigi‑Sierra et al., 2022). In 2015, approximately 3000 Murciano‑Granadina goats were imported from Spain into Kerman Province in southern Iran, a region with tropical climate conditions. This initiative aimed to enhance production efficiency in rural goat flocks and ultimately improve the livelihoods of local dairy farmers. These flocks were traditionally managed under low‑input, low‑output production systems.

In dairy animals, several traits, such as milk yield, milk composition (particularly fat and protein content), and somatic cell score, play key roles in determining overall farm profitability (Godden et al., 2001). Consequently, understanding both the genetic and non‑genetic factors influencing these traits is essential for designing effective breeding programs. Mokhtari et al. (2025) investigated the genetic and phenotypic characteristics of milk, fat, and protein yields in first-parity Murciano-Granadina goats using a traditional model that did not account for parental imprinting effects. Regarding imprinting effects on milk production traits in livestock, only one study has been reported. In that work, Ghafouri-Kesbi et al. (2025) estimated genetic parameters for milk production traits in Iranian Holstein cows using models that incorporated both maternal and paternal imprinting effects.

However, to the best of our knowledge, no previous studies have examined parental imprinting effects on milk production traits in goats. Therefore, the present study was conducted to evaluate the influence of parental imprinting on milk yield and composition traits across the first three lactations of the Murciano-Granadina breed.

## 2 Materials and methods

### 2.1 Pedigree and data

The pedigree used in this study comprised 35,121 Murciano-Granadina goats descended from 937 sires and 11,328 dams. Pedigree preparation for genetic analyses was performed using the CFC program developed by Sargolzaei et al. (2006). Monitoring the pedigree structure (Table 1) showed that 89.81% of animals had both parents identified, 0.63% had only one known parent, and 9.56% had neither parent recorded. Among all registered animals, 34.92% had produced offspring, whereas 65.08% had no progeny. The mean inbreeding coefficient across all animals was 0.29%, while the average among inbred individuals was 4.07%.

**Table 1:** Pedigree structure of the population of the Murciano-Granadina goat.

Item	Numbers
Total individuals	35,121
Total inbred individuals	2484
Total sires	937
Total dams	11,328
Individuals with progeny	12,265
Individuals with no progeny	22,856
Individuals with both known parents	31,543
Individuals with both unknown parents	3358
Individuals with one unknown parent	220
Mean inbreeding coefficients in all individuals (%)	0.29
Mean inbreeding coefficients in the inbred individuals (%)	4.07
Maximum of inbreeding coefficient (%)	31.25
Minimum of inbreeding coefficient (%)	0.10

The original dataset consisted of test‑day records for milk yield, fat percentage, protein percentage, and somatic cell count collected during the first three lactations of Murciano‑Granadina goats between 2017 and 2024 at a private dairy farm, with an average of 2000 lactating goats per year, in Ghale‑Ganj, located in the southern area of Kerman Province, Iran. In the first lactation, the unedited dataset included 45,870 test‑day milk‑yield records from 7003 goats; 9740 test‑day fat‑ and protein‑percentage records from 2653 goats; and 9083 somatic cell count (SCC) records from 3174 goats. For the second lactation, the dataset comprised 35,155 milk‑yield records from 4902 goats; 8714 fat‑ and protein‑percentage records from 2050 goats; and 7817 SCC records from 2653 goats. In the third lactation, there were 23,193 milk‑yield records from 3314 goats; 3693 fat‑ and protein‑percentage records from 1289 goats; and 2984 SCC records from 1248 goats.

For each lactation, test‑day records for milk yield, fat percentage, protein percentage, and SCC were restricted to goats with measurements taken between 5 and 275 days in milk, with sampling intervals of approximately 30 days. Animals with fewer than three or more than nine test‑day records were excluded from the dataset.

### 2.2 Flock management

Newborns were weighed, tagged for identification, and recorded for sex, birth type, and parental information. Weaning occurred at approximately 80 days of age. Using a mating ratio of one buck to 15 goats, maiden goats were bred to fertile bucks at around 11 months of age, when they reached an average body weight of 25 kg (Mokhtari et al., 2024). The dairy goats were fed a ration consisting of commercial concentrate (1350 g/day per goat), alfalfa hay (250 g/day per goat), wheat straw (25 g/day per goat), corn silage (1000 g/day per goat), and sugar beet pulp (1000 g/day per goat). The concentrate mixture included 210 g/kg barley, 400 g/kg corn, 225 g/kg soybean and rapeseed meals, 85 g/kg meat and fat powder, 77 g/kg vitamin-mineral premix, and 3 g/kg salt. Concentrate and hay allowances were adjusted according to age and production stage. Fresh water was available ad libitum. Veterinary care, including antiparasitic treatments and vaccination against enterotoxaemia, foot‑and‑mouth disease, goat pox, and brucellosis, was provided according to the farm’s health management program (Ehsaninia et al., 2025).

### 2.3 Studied traits

To edit raw data, test-day records of milk yield, fat yield, protein yield, and somatic cell count (SCC) were limited to goats for which measurements were taken between 5 −275 days in milk, with consecutive sampling intervals of approximately 30 days. Goats with fewer than three test-day records and more than nine test-day records were excluded from the dataset. The traits analyzed in each lactation were total milk yield (MY), average fat percentage (FP), average protein percentage (PP), and average somatic cell count (SCC). All traits were standardized to a 275‑day lactation period. Total milk yield for each lactation was estimated using the centering data method (Ruiz et al., 2000), also known as Fleischmann’s method, according to the following formula:

$$MY_{i}=\;P_{1}\;\times \;D_{1}+\;\sum_{i=2}^{k}\frac{P_{j}+P_{j-1}}{2}\;\times D_{j}+\;P_{k+1}\;\times \;15$$

where MYᵢ denotes the milk yield in the i‑th lactation (i = 1–3); D₁ is the interval between kidding and the first test‑day record; Pᵢ is the milk yield of the i‑th test‑day record; Dⱼ represents the interval between test‑day j and test‑day (j + 1) (for j = 1,…,k); and 15 is the assumed number of days between the final test‑day and drying‑off. By aggregating individual test‑day measurements into a single cumulative value and conducting genetic analyses on this per‑animal total, a more comprehensive indicator of overall milk‑yield performance is obtained for each animal. Because SCC values were right‑skewed, they were transformed to somatic cell score (SCS) to approximate a normal distribution, using the following transformation (Valencia‑Posadas et al., 2022):

$$SCS=\;log_{2}\;\left(\frac{SCC}{100000}\right)+3$$

For SCS, FP, and PP, each doe’s lactation value was calculated as the average of monthly test‑day records collected up to 275 days in milk. To detect and exclude potential outliers, records for each trait were restricted to the range of the mean ± 3 standard deviations. Descriptive statistics for all traits are presented in Table 2.

**Table 2:** Descriptive statistics for the investigated traits in the Murciano-Granadina goat breed.

Trait ¥	No. of records	Mean	S.D.	C.V. (%)	Min.	Max
MY1 (kg)	6696	375.21	126.37	33.68	100.32	898.60
MY2 (kg)	4611	432.06	140.90	32.61	100.80	884.35
MY3 (kg)	2975	438.47	156.77	35.75	101.40	898.90
FP1 (%)	2579	4.99	0.75	15.03	2.38	9.51
FP2 (%)	2028	4.61	0.85	18.44	2.08	9.78
FP3 (%)	1165	5.09	0.66	12.97	3.03	8.98
PP1 (%)	2579	3.48	0.41	11.78	1.29	7.77
PP2 (%)	2028	3.32	0.45	13.55	1.77	9.90
PP3 (%)	1165	3.60	0.35	9.72	2.46	6.10
SCS1	3065	4.90	1.56	31.84	1.11	9.39
SCS2	2662	4.76	1.54	32.35	1.13	9.40
SCS3	1033	5.45	1.32	24.22	2.19	8.74

¥ MYi: total milk yield in the i^th^ lactation period, FPi: average fat percentage in the i^th^ lactation period, PPi: average protein percentage in the i^th^ lactation period, SCSi: average somatic cell score in the i^th^ lactation period. i: 1, 2, and 3, which stand for the first, second, and third lactation periods, respectively.

### 2.4 Statistical analysis

To determine the significant fixed effects for inclusion in the genetic analysis models, preliminary analyses were conducted using the General Linear Model (GLM) procedure in SAS 9.1 (Statistical Analysis System (SAS), 2004). The fixed effects considered for all traits included kidding year (seven levels: 2018–2024), kidding season (four levels: spring, summer, autumn, winter), kidding type (single or twin), and kidding age. Kidding age was modeled with two levels per lactation: 1‑ and 2‑year‑old goats for first‑lactation traits, 2‑ and 3‑year‑old goats for second‑lactation traits, and 3‑ and 4‑year‑old goats for third‑lactation traits. Mean comparisons among levels of each fixed effect were performed using the Tukey-Kramer test and by considering a p-value of < 0.05 as the significance level.

Genetic analyses for studying the influence of imprinting effects on the investigated traits were performed under four univariate animal models, as follows:

**Table:** 

$y=\mathrm{Xb}+Z_{1}a+e$	**Model 1**
$y=\mathrm{Xb}+Z_{1}a+Z_{2}\mathrm{pt}+e$	**Model 2**
$y=\mathrm{Xb}+Z_{1}a+Z_{3}\mathrm{mt}+e$	**Model 3**
$y=\mathrm{Xb}+Z_{1}a+Z_{2}\mathrm{mt}+Z_{3}\mathrm{pt}+e$	**Model 4**

where, **y** represents a vector of records for the studied traits; **b, a, pt,** and **mt** are vectors of fixed, direct genetic, paternally and maternally imprinted effects, respectively. The matrices **X, Z_1_, Z_2_**, and **Z_3_** are design matrices that associate records with the fixed, direct genetic, paternally imprinted, and maternally imprinted effects, respectively, in vector **y**.

It was assumed that **a** ∼ N (0, **A**$\sigma_{a}^{2}$), **mt** ∼ N (0, **G**$\sigma_{mt}^{2}$), and **pt** ∼ N (0, **G**$\sigma_{pt}^{2}$), and **e** ∼ N (0, **I**$\sigma_{e}^{2}$). In addition, **A** is the numerator relationship matrix, and **G** is the gametic relationship matrix. The inverse of the gametic relationship matrix (**G**^-1^) was used as the supplied covariance matrix in the model with imprinting effects. The matrix **G** was calculated according to the algorithm developed by Tier and Meyer (2012). The investigated models were compared by applying the Akaike^’^s Information Criterion (AIC) (Akaike, 1974). The AIC was computed as follows:

AIC=−2LogL+2p

where Log L is the maximized log-likelihood, and p denotes the number of parameters estimated by the model. Correlations between traits were estimated using bivariate analyses, in which the best-fitting univariate model for each trait was applied. Genetic analyses for all traits were performed using the WOMBAT software package (Meyer, 2013).

### 2.5 Accuracy of models and ranking of animals

The accuracy of the predicted breeding value for each animal i (Accuracy _i_) was calculated by the following formula (Hayes et al., 2009):

$$Accuracy{\;}_{i}=\sqrt{1-(\frac{SE_{i}^{2}}{\sigma_{a}^{2}}})$$

In which SE_i_ is the standard error of the predicted breeding value of the i^th^ animal, and $\sigma_{a}^{2}$ is the additive genetic variance.

Additionally, for traits in which imprinting effects were found to be important, Spearman’s rank‑correlation coefficients between predicted breeding values under the different models were calculated using all animals, as well as the top 50%, 10%, and 1% of individuals. This enabled an assessment of how model choice influenced the ranking of animals based on their predicted breeding values.

## 3 Results

### 3.1 Descriptive statistics for the studied traits

As presented in Table 2, the lowest average milk yield was in the first lactation and the highest one in the third lactation period. For the other traits studied, the lowest average was obtained in the second lactation period and the highest in the third lactation period. Overall, coefficients of variation (CV) for milk yield (MY1, MY2, MY3) and somatic cell score (SCS1, SCS2, SCS3) traits were higher than those for fat (FP1, FP2, FP3) and protein percentages (PP1, PP2, PP3) traits. Among the milk‑yield traits, the highest CV occurred in the third lactation and the lowest in the second. In contrast, for the remaining traits, the lowest CV was observed in the third lactation and the highest in the second.

### 3.2 Fixed effects

Least‑square means for milk yield (MY1, MY2, MY3) and fat percentage (FP1, FP2, FP3) traits across the evaluated fixed effects are presented in Table 3, while the corresponding means for protein percentage (PP1, PP2, PP3) and somatic cell score (SCS1, SCS2, SCS3) are shown in Table 4. Both kidding year and kidding season had significant effects on all traits (p < 0.01). Kidding age significantly influenced MY1 and MY2 (p < 0.01) but showed no significant effect on the remaining traits (p > 0.05). Kidding type significantly affected all milk‑yield traits (MY1–MY3; p < 0.01), whereas no significant effects were observed for the other traits (p > 0.05).

**Table 3:** Least square means (± S.E.) for the milk yield and fat percentage in the first three lactation periods of the Murciano-Granadina goat.

Effect	Trait ¥					
	MY1 (kg)	MY2 (kg)	MY3 (kg)	FP1 (%)	FP2 (%)	FP3 (%)
Kidding year	**	**	**	**	**	**
2018	432.99 ± 3.16 ^a^	502.94 ± 3.45 ^a^	547.58 ± 10.70 ^a^	4.23 ± 0.07 ^c^	4.12 ± 0.07 ^d^	-
2019	365.29 ± 5.61 ^c^	460.89 ± 3.86 ^b^	474.23 ± 5.07 ^b^	4.67 ± 0.06 ^b^	4.43 ± 0.03 ^c^	-
2020	399.90 ± 5.83 ^b^	479.06 ± 5.88 ^ab^	443.96 ± 7.34 ^c^	4.57 ± 0.06 ^b^	4.04 ± 0.07 ^d^	4.48 ± 0.06 ^c^
2021	424.56 ± 4.24 ^a^	450.41 ± 7.65 ^b^	463.01 ± 9.64 ^bc^	4.15 ± 0.08 ^c^	4.93 ± 0.05 ^b^	4.99 ± 0.04 ^b^
2022	395.98 ± 4.01 ^b^	467.14 ± 5.67 ^ab^	399.56 ± 7.57 ^d^	5.14 ± 0.03 ^a^	4.99 ± 0.04 ^b^	5.04 ± 0.05 ^ab^
2023	353.04 ± 3.34 ^c^	398.03 ± 4.86 ^c^	315.75 ± 6.37 ^e^	5.10 ± 0.03 ^a^	5.19 ± 0.05 ^a^	5.09 ± 0.09 ^a^
2024	277.74 ± 3.34 ^d^	325.68 ± 4.50 ^d^	-	4.83 ± 0.04 ^ab^	5.43 ± 0.07 ^a^	-
Kidding season	**	**	**	**	**	**
Spring	382.80 ± 3.08 ^b^	435.69 ± 4.78 ^b^	435.50 ± 6.88 ^a^	4.64 ± 0.06 ^b^	4.71 ± 0.16 ^b^	4.81 ± 0.11 ^c^
Summer	347.25 ± 3.75 ^c^	417.24 ± 5.38 ^c^	427.94 ± 7.08 ^b^	4.95 ± 0.06 ^a^	4.89 ± 0.16 ^a^	5.27 ± 0.10 ^a^
Autumn	389.00 ± 2.64 ^b^	450.39 ± 4.51 ^ab^	449.25 ± 5.45 ^a^	4.63 ± 0.07 ^b^	4.72 ± 0.16 ^b^	5.04 ± 0.10 ^b^
Winter	414.95 ± 3.40 ^a^	459.04 ± 3.76 ^a^	450.04 ± 5.08 ^a^	4.56 ± 0.06 ^b^	4.69 ± 0.15 ^b^	4.68 ± 0.11 ^c^
Kidding age	**	**	ns	ns	ns	ns
1-year	364.47 ± 1.92 ^b^	-	-	4.75 ± 0.06 ^a^	-	-
2-year	392.53 ± 2.79 ^a^	426.04 ± 2.99 ^b^	-	4.73 ± 0.06 ^a^	4.78 ± 0.16 ^a^	-
3-year	-	445.14 ± 4.26 ^a^	438.19 ± 3.50 ^a^	-	4.78 ± 0.16 ^a^	5.05 ± 0.09 ^a^
4-year	-	-	443.17 ± 6.15 ^a^	-	-	4.95 ± 0.10 ^a^
Kidding type	**	**	**	ns	ns	ns
Single	369.12 ± 2.19 ^b^	413.38 ± 3.25 ^a^	420.58 ± 2.63 ^a^	4.85 ± 0.04 ^a^	4.46 ± 0.11 ^a^	5.08 ± 0.09 ^a^
Twin	409.43 ± 4.25 ^a^	441.19 ± 4.69 ^b^	452.15 ± 3.85 ^b^	4.83 ± 0.05 ^a^	4.45 ± 0.11 ^a^	5.05 ± 0.13 ^a^

¥ MYi: total milk yield in the i^th^ lactation period; FPi: average fat percentage in the i^th^ lactation period. i: 1, 2, and 3, which stand for the first, second, and third lactation period, respectively. Means with similar letters in each sub-class within a column do not differ from each other at P > 0.05; ns: not significant. Means with different letters in each sub-class within a column differ from each other at * P < 0.05 and ** P < 0.01.

**Table 4:** Least square means (± S.E.) for the milk protein percentage and somatic cell scores in the first three lactation periods of the Murciano-Granadina goat.

Effect	Trait ¥					
	PP1	PP2	PP3	SCS1	SCS2	SCS3
Kidding year	**	**	**	**	**	**
2018	3.48 ± 0.05 ^b^	3.39 ± 0.06 ^c^	-	4.48 ± 0.07 ^b^	4.46 ± 0.05 ^c^	-
2019	3.34 ± 0.03 ^bc^	3.21 ± 0.04 ^d^	-	4.27 ± 0.10 ^c^	4.50 ± 0.06 ^c^	-
2020	2.86 ± 0.05 ^d^	3.49 ± 0.07 ^c^	3.49 ± 0.08 ^b^	4.49 ± 0.09 ^b^	4.90 ± 0.10 ^b^	-
2021	3.69 ± 0.02 ^a^	3.64 ± 0.02 ^b^	3.64 ± 0.02 ^a^	5.16 ± 0.08 ^a^	5.57 ± 0.10 ^a^	5.61 ± 0.06 ^a^
2022	3.48 ± 0.01 ^b^	3.53 ± 0.03 ^bc^	3.53 ± 0.03 ^b^	5.26 ± 0.06 ^a^	5.16 ± 0.09 ^a^	5.13 ± 0.06 ^b^
2023	3.26 ± 0.02 ^c^	3.18 ± 0.05 ^d^	3.18 ± 0.05 ^c^	5.36 ± 0.08 ^a^	5.25 ± 0.09 ^a^	5.06 ± 0.13 ^b^
2024	3.73 ± 0.05 ^a^	3.85 ± 0.05 ^a^	-	4.62 ± 0.03 ^b^	5.15 ± 0.10 ^a^	-
Kidding season	**	**	**	**	**	**
Spring	3.39 ± 0.03 ^b^	3.50 ± 0.06 ^b^	3.38 ± 0.03 ^b^	4.87 ± 0.08 ^a^	5.16 ± 0.09 ^a^	5.56 ± 0.07 ^a^
Summer	3.54 ± 0.03 ^a^	3.64 ± 0.05 ^a^	3.55 ± 0.02 ^a^	4.97 ± 0.08 ^a^	5.18 ± 0.10 ^a^	5.58 ± 0.10 ^a^
Autumn	3.29 ± 0.03 ^c^	3.44 ± 0.05 ^b^	3.43 ± 0.03 ^a^	4.59 ± 0.09 ^b^	4.84 ± 0.09 ^b^	5.22 ± 0.07 ^b^
Winter	3.19 ± 0.03 ^d^	3.27 ± 0.05 ^c^	3.42 ± 0.04 ^b^	4.58 ± 0.08 ^b^	4.82 ± 0.08 ^b^	4.93 ± 0.09 ^c^
Kidding age	ns	ns	ns	ns	ns	ns
1-year	3.38 ± 0.03 ^a^	-	-	4.97 ± 0.06 ^a^	-	-
2-year	3.36 ± 0.03 ^b^	3.51 ± 0.06 ^a^	-	4.94 ± 0.07 ^b^	4.99 ± 0.06 ^a^	-
3-year	-	3.49 ± 0.05 ^a^	3.45 ± 0.05 ^a^	-	5.00 ± 0.08 ^a^	5.24 ± 0.05 ^a^
4-year	-	-	3.42 ± 0.05 ^a^	-	-	5.30 ± 0.07 ^a^
Kidding type	ns	ns	ns	ns	ns	ns
Single	3.43 ± 0.05 ^a^	3.29 ± 0.04 ^a^	3.48 ± 0.06 ^a^	4.83 ± 0.07 ^a^	4.69 ± 0.06 ^a^	5.29 ± 0.06 ^a^
Twin	3.42 ± 0.05 ^a^	3.27 ± 0.05 ^a^	3.49 ± 0.06 ^a^	4.86 ± 0.08 ^a^	4.71 ± 0.05 ^a^	5.33 ± 0.07 ^a^

¥ PPi: average milk protein percentage in the i^th^ lactation period; SCSi: average milk somatic cell score in the i^th^ lactation period. i: 1, 2, and 3, which stand for the first, second, and third lactation period, respectively. Means with similar letters in each sub-class within a column do not differ from each other at P > 0.05; ns: not significant. Means with different letters in each sub-class within a column differ from each other at * P < 0.05 and ** P < 0.01.

### 3.3 Model comparisons

The results of comparisons of investigated animal models based on AIC values are presented in Table 5 for MY1, MY2, MY3, FP1, FP2, and FP3, and in Table 6 for PP1, PP2, PP3, SCS1, SCS2, and SCS3. Parental imprinting effects were important only for MY1, MY2, FP1, and FP2. Model 2, which contains direct additive genetic and paternal imprinting effects, was detected as the best model for MY1, FP1, and FP2. Model 4, which contains direct additive genetic, paternal imprinting, and maternal imprinting effects, was the best model for MY2. For other studied traits, Model 1, which contains direct genetic effects, was the best model for estimating variance components and genetic parameters.

**Table 5:** Estimates of variance components and genetic parameters for the milk yield and fat percentage in the first three lactation periods of the Murciano-Granadina goat; the best model is shown in bold.

Trait ¥	Model ¥¥	$\sigma_{a}^{2}$	$\sigma_{pt}^{2}$	$\sigma_{mt}^{2}$	$\sigma_{e}^{2}$	$\sigma_{p}^{2}$	$h^{2}$	$pt^{2}$	$mt^{2}$	AIC	Accuracy
MY1	Model 1	1743.170	-	-	10,543.800	12,286.970	0.14±0.02	-	-	69,590.76	0.55
	**Model 2**	**974.855**	**851.130**	**-**	**10,508.195**	**12,334.180**	**0.08±0.03**	**0.07±0.02**	**-**	**69,583.56**	**0.58**
	Model 3	1527.88	-	0.002	10,651.478	12,179.36	0.13±0.02	-	0.00±0.02	69,592.76	0.56
	Model 4	974.869	863.39	0.026	10,495.925	12,334.21‬0	0.08±0.03	0.07±0.02	0.00±0.03	69,585.56‬	0.56
MY2	Model 1	2083.480	-	-	14,330.700	16,414.180	0.13±0.03	-	-	49,173.06	0.50
	Model 2	1904.660	225.080	-	14,300.900	16,430.640	0.12±0.03	0.01±0.02	-	49,174.48	0.51
	Model 3	1572.930	-	1115.730	13,690.600	16,379.26‬0	0.10±0.03	-	0.07±0.03	49,171.38	0.52
	**Model 4**	**1080.310**	**380.636**	**1325.610**	**13,600.804**	**16,387.36**	**0.07±0.03**	**0.02±0.01**	**0.08±0.02**	**49,170.22**	**0.56**
MY3	**Model 1**	**1368.470**	**-**	**-**	**18,483.200**	**19,851.670**	**0.07±0.03**	**-**	**-**	**32,347.44**	**0.47**
	Model 2	1338.810	108.650	-	18,406.240	19,853.7‬00	0.07±0.03	0.00±0.03	-	32,353.58	0.45
	Model 3	1339.120	-	107.120	18,514.500	19,960.740	0.07±0.03	-	0.00±0.05	32,353.58	0.45
	Model 4	975.640	8.970	7.320	18,409.160	19,401.09	0.00±0.05	0.05±0.03	0.00±0.06	32,352.96	0.44
FP1	Model 1	0.116	-	-	0.373	0.489	0.24±0.05	-	-	574.08	0.21
	**Model 2**	**0.087**	**0.035**	**-**	**0.369**	**0.491**	**0.18±0.06**	**0.07±0.03**	**-**	**573.96**	**0.26**
	Model 3	0.096	-	0.018	0.374	0.488	0.20±0.06	-	0.04±0.03	575.68	0.24
	Model 4	0.063	0.041	0.029	0.356	0.489	0.13±0.04	0.08±0.03	0.06±0.04	575.32	0.21
FP2	Model 1	0.071	-	-	0.515	0.586	0.12±0.04	-	-	1219.44	0.22
	**Model 2**	**0.032**	**0.059**		**0.497**	**0.588**	**0.05±0.02**	**0.10±0.04**	**-**	**1209.82**	**0.26**
	Model 3	0.051	-	0.002	0.537	0.590	0.12±0.04	-	0.00±0.04	1221.44	0.25
	Model 4	0.001	0.059	0.010	0.518	0.678	0.00±0.06	0.09±0.04	0.02±0.06	1211.72	0.21
FP3	**Model 1**	**0.081**	**-**	**-**	**0.340**	**0.421**	**0.19±0.04**	**-**	**-**	**139.68**	**0.21**
	Model 2	0.003	0.001	-	0.401	0.405	0.00±0.04	0.00±0.03	-	141.98	0.16
	Model 3	0.001	-	0.014	0.394	0.409	0.00±0.03	-	0.03±0.04	141.20	0.19
	Model 4	0.001	0.001	0.021	0.366	0.389	0.02±0.07	0.02±0.08	0.05±0.07	143.42	0.15

¥ MYi: total milk yield in the i^th^ lactation period, FPi: average fat percentage in the i^th^ lactation period. i: 1, 2, and 3, which stand for the first, second, and third lactation period, respectively.

¥¥ Model 1: included direct additive genetic effects; Model 2: included direct additive genetic and paternal imprinting effects; Model 3: included direct additive genetic and maternal imprinting effects; Model 4: included direct additive genetic, paternal, and maternal imprinting effects. $\sigma_{a}^{2}$: additive genetic variance,$\;\sigma_{pt}^{2}$: paternal imprinting variance, $\sigma_{mt}^{2}$: maternal imprinting variance, $\sigma_{e}^{2}$: residual variance, $\sigma_{p}^{2}$: phenotypic variance, $h^{2}$: direct heritability, $mt^{2}$: maternal imprinting heritability, $pt^{2}$: paternal imprinting heritability.

**Table 6:** Estimates of variance components and genetic parameters for the protein percentage and somatic cell score in the first three lactation periods of the Murciano-Granadina goat; the best model is shown in bold.

Trait ¥	Model ¥¥	$\sigma_{a}^{2}$	$\sigma_{pat}^{2}$	$\sigma_{mat}^{2}$	$\sigma_{e}^{2}$	$\sigma_{p}^{2}$	$h^{2}$	$pt^{2}$	$mt^{2}$	AIC	Accuracy
PP1	**Model 1**	**0.010**	**-**	**-**	**0.134**	**0.144**	**0.07±0.02**	**-**	**-**	**−1926.89**	**0.35**
	Model 2	0.002	0.002	-	0.135	0.139	0.01±0.04	0.01±0.03	-	−1921.66‬	0.29
	Model 3	0.008	-	0.001	0.136	0.145	0.05±0.03	-	0.00±0.06	−1917.67	0.32
	Model 4	0.001	0.003	0.001	0140	0.145	0.01±0.07	0.02±0.05	0.01±0.07	−1920.37	0.26
PP2	**Model 1**	**0.067**	**-**	**-**	**0.128**	**0.195**	**0.34±0.05**	**-**	**-**	**−1705.57**	**0.35**
	Model 2	-	-	-	-	-	-	-	-	NC	0.27
	Model 3	0.081	-	0.001	0.135	0.217	0.37±0.07	-	0.00±0.06	−1690.74	0.31
	Model 4	-	-	-	-	-	-	-	-	NC	0.25
PP3	**Model 1**	**0.013**	**-**	**-**	**0.092**	**0.105**	**0.12±0.04**	**-**	**-**	**−1425.49‬**	**0.29**
	Model 2	0.002	0.011	-	0.099	0.112	0.01±0.05	0.10±0.04	-	−1423.77‬	0.22
	Model 3	0.006	-	0.001	0.107	0.114	0.05±0.09	-	0.01±0.05	−1418.89	0.24
	Model 4	0.001	0.009	0.001	0.102	0.113	0.01±0.09	0.08±0.05	0.01±0.08	−1423.86‬	0.21
SCS1	**Model 1**	**0.239**	**-**	**-**	**2.031**	**2.270**	**0.10±0.03**	**-**	**-**	**5598.79‬**	**0.42**
	Model 2	0.043	0.179	-	2.060	2.282	0.02±0.04	0.08±0.03	-	5599.82‬	0.35
	Model 3	0.079	-	0.066	2.143	2.288	0.03±0.03	-	0.03±0.01	5609.50	0.36
	Model 4	0.088	0.212	0.409	2.060	2.769	0.03±0.08	0.08±0.04	0.15±0.08	5654.06	0.32
SCS2	**Model 1**	**0.433**	**-**	**-**	**1.713**	**2.146**	**0.20±0.04**	**-**	**-**	**4659.65**	0.39
	Model 2	0.109	0.227	-	1.807	2.143	0.05±0.02	0.10±0.03	-	4667.00	0.35
	Model 3	0.231	-	0.005	1.909	2.145	0.11±0.05	-	0.00±0.05	4676.53	0.33
	Model 4	0.066	0.244	0.059	1.773	2.142	0.03±0.01	0.11±0.05	0.03±0.01	4660.91‬	0.30
SCS3	**Model 1**	**0.073**	**-**	**-**	**0.746**	**0.819**	**0.09±0.02**	**-**	**-**	**844.70‬**	**0.25**
	Model 2	0.065	0.001	-	0.752	0.818	0.08±0.07	0.00±0.09	-	853.47	0.22
	Model 3	0.068	-	0.001	0.752	0.821	0.09±0.07	-	0.00±0.09	853.47	0.22
	Model 4	0.070	0.001	0.001	0.751	0.823	0.08±0.06	0.00±0.00	0.00±0.09	855.51	0.21

¥ PPi: average protein percentage in the i^th^ lactation period; SCSi: average somatic cell score in the i^th^ lactation period. i: 1, 2, and 3, which stand for the first, second, and third lactation period, respectively.

¥¥ Model 1: included direct additive genetic effects; Model 2: included direct additive genetic and paternal imprinting effects; Model 3: included direct additive genetic and maternal imprinting effects; Model 4: included direct additive genetic, paternal, and maternal imprinting effects. $\sigma_{a}^{2}$: additive genetic variance,$\;\sigma_{pt}^{2}$: paternal imprinting variance, $\sigma_{mt}^{2}$: maternal imprinting variance, $\sigma_{e}^{2}$: residual variance, $\sigma_{p}^{2}$: phenotypic variance, $h^{2}$: direct heritability, $mt^{2}$: maternal imprinting heritability, $pt^{2}$: paternal imprinting heritability. NC: No convergence.

### 3.4 Variance components and heritability estimates

Estimates of variance components and heritability for MY1, MY2, MY3, FP1, FP2, and FP3 under the four animal models are presented in Table 5, and those for PP1, PP2, PP3, SCS1, SCS2, and SCS3 are shown in Table 6. Paternal imprinting effects accounted for 7%, 2%, 7%, and 10% of the phenotypic variance in MY1, MY2, FP1, and FP2, respectively. Incorporating paternal imprinting into the model reduced the direct heritability estimates for MY1 (from 0.14 ± 0.02 to 0.08 ± 0.03), FP1 (from 0.24 ± 0.05 to 0.18 ± 0.06), and FP2 (from 0.12 ± 0.04 to 0.05 ± 0.02) (Table 5). For MY2, inclusion of both paternal and maternal imprinting effects lowered the direct heritability estimate from 0.13 ± 0.03 (Model 1) to 0.07 ± 0.03 (Model 2). Under the best‑fitting univariate model, direct heritability estimates ranged from 0.05 ± 0.02 for FP2 (Table 5) to 0.34 ± 0.05 for PP2 (Table 6), indicating low to moderate heritability across traits. Paternal imprinting heritability (pt²) was low, ranging from 0.02 ± 0.01 for MY2 to 0.10 ± 0.04 for FP2. A similarly low maternal imprinting heritability (mt²) of 0.08 ± 0.02 was estimated for MY2.

### 3.5 The parental imprinting effects on the accuracy of models and ranking of animals

The average accuracies of the predicted breeding values of animals for MY1 (0.55 vs. 0.58), MY2 (0.56 vs. 0.50), FP1 (0.26 vs. 0.21), and FP2 (0.26 vs. 0.22) traits under the model including parental imprinting effects were higher than those of the model excluding parental imprinting effects (Table 5). For the traits influenced by parental imprinting (MY1, MY2, FP1, and FP2), Spearman’s rank correlations between estimated breeding values from Model 1, which excluded parental imprinting effects, and the best-fitting model including imprinting effects, calculated for all animals, the top 50%, the top 10%, and the top 1%, are presented in Table 7. All correlations were positive and highly significant (p < 0.01), remaining close to unity when all animals were considered but decreasing as the analysis focused on higher‑ranking subsets, particularly the top 1%.

**Table 7:** Spearman’s rank correlation between predicted breeding values of animals between models that included and excluded parental imprinting effects.

Trait ¥	All animals	50% top-ranked	10% top-ranked	1% top ranked
MY1	0.98 ⁎⁎	0.92 ⁎⁎	0.82 ⁎⁎	0.73 ⁎⁎
MY2	0.99 ⁎⁎	0.94 ⁎⁎	0.87 ⁎⁎	0.75 ⁎⁎
FP1	0.98 ⁎⁎	0.95 ⁎⁎	0.85 ⁎⁎	0.81 ⁎⁎
FP2	0.93 ⁎⁎	0.89 ⁎⁎	0.71 ⁎⁎	0.68 ⁎⁎

¥ MY1: total milk yield in the first lactation period, MY2: total milk yield in the second lactation period, FP1: average fat percentage in the first lactation period, FP2: average fat percentage in the second lactation period.

⁎⁎ P < 0.01.

### 3.6 Correlation estimates

Estimated correlations among the studied traits are presented in Supplementary Table 1. Overall, genetic correlations were larger in magnitude than the phenotypic, residual, and paternal‑imprinting correlations (the latter reported only for traits significantly affected by paternal imprinting).

Fig. 1 showed a visual representation of genetic and phenotypic correlations among the investigated traits. Genetic correlations among the same traits across lactations were positive and ranged from 0.81 ± 0.10 for MY1–MY2 to 0.91 ± 0.25 for MY2-MY3, from 0.11 ± 0.01 for FP1-FP3 to 0.77 ± 0.04 for FP2-FP3, from 0.41 ± 0.05 for PP1-PP3 to 0.54± 0.05 for PP1-PP2, and from 0.23 ± 0.03 for SCS1-SCS2 to 0.26 ± 0.04 for SCS1-SCS3. Genetic correlations among milk composition traits were lower than the correlations observed among milk-yield traits. Genetic correlations among the studied traits within lactation periods ranged from −0.63± 0.14 (MY1-FP1) to 0.92± 0.25 (FP1-PP1) in the first lactation period, from −0.71± 0.23 (MY2-PP2) to 0.88± 0.07 (PP2-SCS2) in the second lactation period, and from −0.69± 0.21 (MY3-FP3) to 0.96± 0.23 (FP3-PP3) in the third lactation period.

**Fig. 1:**
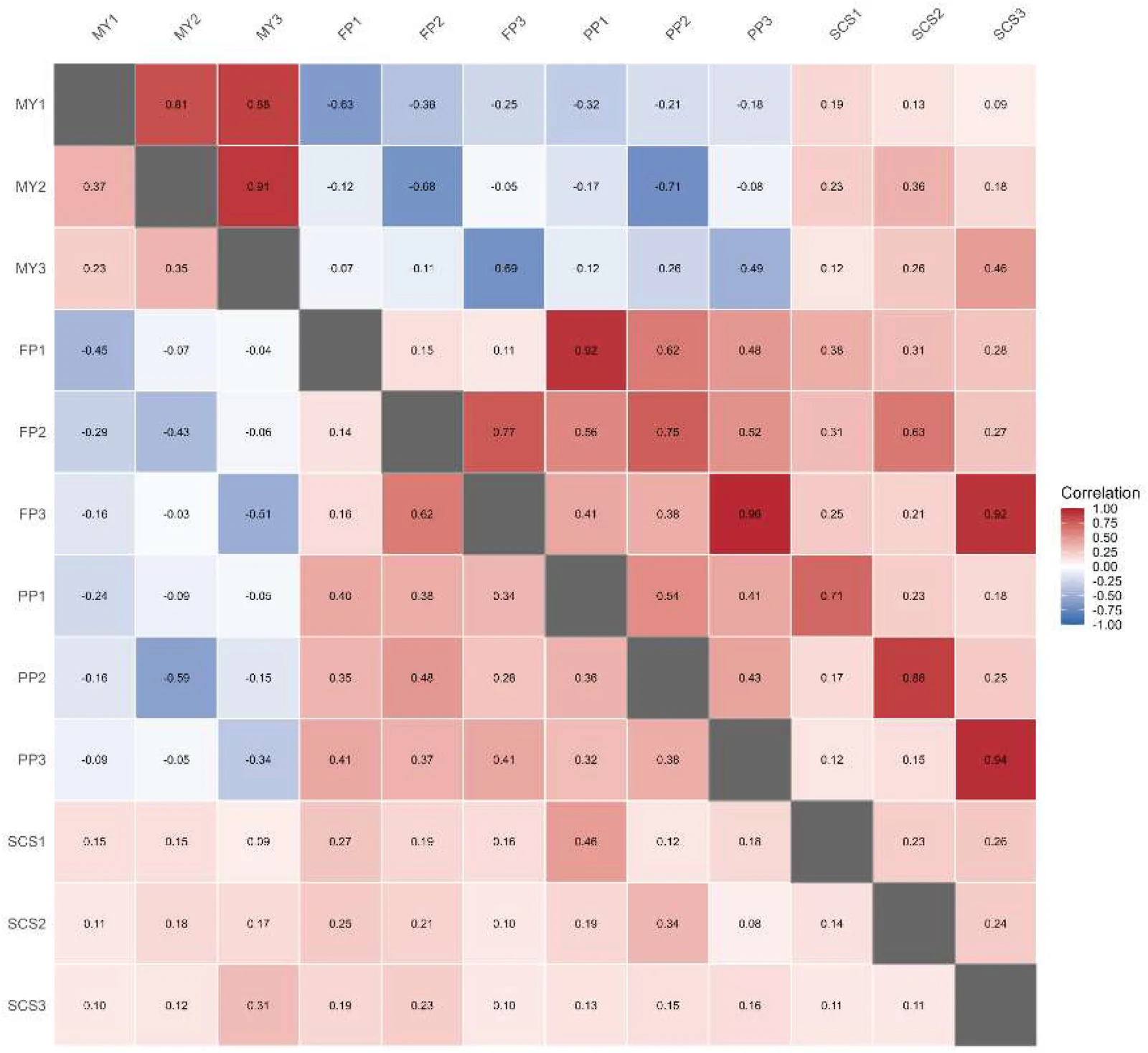
Visual representation of genetic (above the diagonal) and phenotypic (below the diagonal) correlations among the investigated traits.

MYi: total milk yield in the i^th^ lactation period, FPi: average fat percentage in the i^th^ lactation period, PPi: average protein percentage in the i^th^ lactation period, SCSi: average somatic cell score in the i^th^ lactation period. i: 1, 2, and 3, which stand for the first, second, and third lactation periods, respectively.

In general, phenotypic correlations among the same traits across lactations were positive but lower than the corresponding genetic correlations, ranged from 0.23± 0.02 (MY1-MY3) to 0.37± 0.01 (MY1-MY2), from 0.14± 0.03 (FP1-FP2) to 0.62± 0.03 (FP2-FP3), from 0.32± 0.10 (PP1-PP3) to 0.38± 0.03 (PP2-PP3), and from 0.11± 0.03 (SCS1-SCS3 and SCS2-SCS3) to 0.14± 0.02 (SCS1-SCS2). Phenotypic correlations among the studied traits within lactation periods ranged from −0.45± 0.04 (MY1-FP1) to 0.46± 0.02 (PP1-SCS1) in the first lactation period, from −0.59± 0.05 (MY2-PP2) to 0.48± 0.02 (FP2-PP22) in the second lactation period, and from −0.51± 0.06 (MY3-FP3) to 0.41± 0.03 (FP3-PP3) in the third lactation period. In general, the magnitudes and signs of residual correlations among the traits followed similar trends to those of phenotypic correlations. As shown in Supplementary Table 1, paternal‑imprinting correlations ranged from −0.39 ± 0.15 for MY1-FP1 to 0.68 ± 0.13 for MY1-MY2.

## 4 Discussion

### 4.1 Overview of the phenotypic performance

The mean milk‑yield values (MY1, MY2, and MY3) obtained in the present study were lower than those reported by Wiggans and Hubbard (2001) for Alpine (1066 kg), LaMancha (898 kg), Nubian (890 kg), Oberhasli (882 kg), Saanen (1060 kg), and Toggenburg (970 kg) goats. They also reported mean fat percentages of 3.5%, 3.5%, 4.5%, 3.7%, 3.4%, and 3.3% for Alpine, LaMancha, Nubian, Oberhasli, Saanen, and Toggenburg breeds, respectively, values that were lower than the fat‑percentage means recorded for the first three lactations of Murciano‑Granadina goats in this study, which may be justified to some extent by breed differences, undergoing adaptation process of the studied population of the Murciano-Granadina goat and different selection programs. In contrast, Valencia‑Posadas et al. (2022) reported a mean yield of 314.05 kg in Nigerian Dwarf goats, which was lower than the values observed in our population. Similarly, Spehar et al. (2019) reported lower fat (3.47%) and protein (3.10%) percentages in Alpine goats from Croatia. In contrast, Valencia‑Posadas et al. (2022) reported higher fat (6.60%) and protein (4.45%) percentages in Nigerian Dwarf goats. Castaneda‑Bustos et al. (2014) also reported lower mean fat (3.87%) and protein (3.18%) percentages in U.S. dairy goats compared with the corresponding values observed here. The mean SCS values (SCS1, SCS2, and SCS3) in the present study were generally consistent with those reported by Aguirre‑Arroyo et al. (2024) for Nubian (4.88), Alpine (4.88), LaMancha (4.87), Saanen (4.89), and Toggenburg (4.92) goats. They also noted lower SCS means of 2.94 and 4.46 in Nigerian Dwarf and Oberhasli breeds, respectively. The observed differences may be explained by the distinct breeding goals of the breeds compared. The Murciano-Granadina, a Spanish dairy breed synthesized in the south-east of the country, is raised in an area where goat milk is predominantly processed into cheese. Its official breeding program therefore emphasizes milk composition, namely fat, protein, and dry-extract yields, together with udder conformation, rather than on milk volume alone (Delgado et al., 2017). Conversely, Alpine, Saanen, and Toggenburg goats have been selected primarily for milk volume under intensive production systems, which agrees with their greater yields and lower fat percentages (Wiggans & Hubbard, 2001). Moreover, the population studied here descends from animals imported from Spain to a hot region of Iran in 2015, and its lower yields may partly indicate incomplete adaptation to the local climate. Finally, variation in recording methods and in the standardization of lactation yields among studies may also account for part of these differences.

In the present study, the milk‑yield traits (MY1, MY2, and MY3) and somatic cell score traits (SCS1, SCS2, and SCS3) exhibited higher coefficients of variation than the fat‑percentage (FP1, FP2, and FP3) and protein‑percentage traits (PP1, PP2, and PP3). The relatively higher variability in milk yield and somatic cell score than in fat and protein percentages suggests these traits are more phenotypically variable, making them key targets for selection programs aimed at improving production performance and udder health, which may be evaluated by somatic cell score as a relevant measure of mammary gland health (Becker et al., 2020).

### 4.2 The influence of non-genetic effects

The significant effects of year and season of kidding on the studied traits are likely due to seasonal and annual variations in climate, feed availability, and nutritional quality (Selvaggi & Dario, 2015). As shown in Table 3, the lowest least‑squares means for MY1, MY2, and MY3 occurred in summer, whereas the highest values were observed in winter. For MY2, the means in winter and autumn did not differ significantly (p > 0.05), and for MY3, the differences among autumn, winter, and spring were also not significant (p > 0.05). Fat‑percentage traits (FP1, FP2, and FP3) showed the highest least‑squares means in summer. No significant differences were detected among spring, autumn, and winter for FP1 and FP2. However, FP3 was significantly higher in autumn than in both spring and winter (p < 0.01), while the winter–spring difference was not significant (p > 0.05). As shown in Table 4, protein‑percentage traits (PP1, PP2, and PP3) followed a similar seasonal pattern. The highest means for PP1, PP2, and PP3 were recorded in summer. For PP1 and PP2, the lowest means occurred in winter, whereas PP3 did not differ significantly among spring, autumn, and winter (p > 0.05). Seasonal variations in the composition of goat milk are well documented by Saviard et al. (2026). They pointed out that higher milk-fat and milk-protein percentages in goats kidded in the summer season may reflect concentration effects due to reduced milk volume.

Significant effects of kidding season on milk‑yield traits have also been reported in Mexican Saanen goats (Valencia et al., 2002) and Black Bengal goats in Bangladesh (Mahal et al., 2014). In general, milk‑production traits are influenced by multiple factors, including interactions between the animal and environmental temperature, as well as the availability and quality of forage across seasons (Irawati et al., 2020). Although the Murciano‑Granadina goats in this study were managed under an intensive production system, where feed supply is largely controlled, seasonal weather patterns and temperature fluctuations can still affect milk‑production performance (Biswal et al., 2021). Aleena et al. (2018) noted that during periods of heat stress, animals increase their physiological and behavioral adaptive responses to cope with elevated temperatures. These adaptive processes require additional energy, causing animals to divert energy away from productive functions such as milk synthesis and toward maintaining homeostasis (Aleena et al., 2018). Somatic cell score traits (SCS1, SCS2, and SCS3) also reached their highest values in summer, with no significant differences between summer and spring (p > 0.05). The lowest SCS means were observed in winter, and the differences between winter and autumn for SCS1 and SCS2 were not significant (p > 0.05). Mastitis is one of the primary causes of increased somatic cell count in milk of livestock (Oliveira et al., 2025). Becker et al. (2020) noted that rising environmental temperature and the consequent heat stress pose an increased risk for the incidence of mastitis in dairy cows, as it depresses the immune response. Sargeant et al. (1998) reported higher milk somatic cell count in Canadian dairy cows parturiated in the summer compared to other seasons of the year.

Two‑year‑old Murciano‑Granadina goats produced significantly higher MY1 than one‑year‑old goats, and three‑year‑old goats produced significantly higher MY2 than two‑year‑olds (Table 3). These differences are likely related to advancing maturity and the continued development of mammary secretory tissue (Knight & Peaker, 1982). Scholtens et al. (2019) similarly reported significant effects of doe age at kidding on total lactation milk yield in New Zealand dairy goats. Goats that kidded singly had significantly lower least‑squares means for MY1, MY2, and MY3 compared with twin‑kidding goats (p < 0.01). The influence of kidding type is commonly attributed to changes in prepartum endocrine profiles associated with litter size. Hormones produced by the fetoplacental unit, such as progesterone, estrogen, and placental lactogen, play key roles in mammogenesis and lactogenesis (De Louis et al., 1980). Increased fetal and placental mass in multiple pregnancies can therefore enhance prepartum hormonal stimulation, contributing to higher milk yields (Schoknecht et al., 1991). Significant effects of kidding type on milk yield have also been reported in Jonica goats (Selvaggi & Dario, 2015), Jamunapari goats (Rout et al., 2017), Australian commercial dairy goats (Zamuner et al., 2020), and Tunisian local goats (Atoui et al., 2024). These studies consistently attribute the superior milk yield of multiple‑kidding goats to increased hormonal activity, particularly progesterone, placental lactogen, and prolactin, which enhance mammary gland development during gestation.

### 4.3 Imprinting effects, accuracy of the models and ranking of animals based on the estimated breeding values

The detection of paternal imprinting effects for MY1, MY2, FP1, and FP2 suggests that sire‑derived alleles exert imprinting effects on milk yield and milk-fat percentage in the first and second lactation periods of the studied population of the Murciano-Granadina goat breed. The additional maternal imprinting effect observed for MY2 indicates that dam‑derived alleles may also contribute to the influence on milk yield in the second lactation period of the studied population of the Murciano-Granadina goat breed. For milk-protein percentage (PP1–PP3) and somatic cell score (SCS1–SCS3) traits, the best model was the simplest one (direct additive genetic effects only). This indicates that protein synthesis in milk and also somatic cell scores in the first three lactation periods of the studied population of the Murciano-Granadina goat breed are largely governed by additive genetic variation without detectable parent‑of‑origin effects.

The comparison of the model accuracies indicates that incorporating parental imprinting effects improved the accuracy of estimated breeding values for MY1, MY2, FP1, and FP2, suggesting that imprinting effects contribute significantly to the genetic architecture of these traits. This pattern is consistent with previous studies showing that imprinting can influence quantitative traits and that models accounting for imprinting often capture additional variance not explained by additive effects alone (Garcia-Cortes & Sorensen, 2001; de Koning & Haley, 2002). Empirical work in livestock and model organisms has similarly demonstrated that imprinting can affect production traits and improve prediction when appropriately modeled (Wolf et al., 2008).

For MY3, FP3, milk-protein percentage, and somatic cell score traits, the model without imprinting effects yielded higher model accuracies. This outcome aligns with theoretical expectations that imprinting is highly trait-specific and may be negligible for many quantitative traits (Spencer, 2009; Santure & Spencer, 2006). When imprinting variance is absent or minimal, adding imprinting parameters increases model complexity without improving fit, which can reduce predictive performance due to overparameterization (Legarra et al., 2009). Thus, the superior accuracy of the simpler additive model for these traits suggests that their genetic architecture is dominated by additive effects, with little or no contribution from parent-of-origin mechanisms.

Overall, these results highlight that the usefulness of imprinting-aware models depends on the biological relevance of imprinting for each trait. Traits influenced by parent-of-origin effects benefit from models that incorporate imprinting, whereas traits lacking such effects are better predicted by traditional additive models. This trait-specific pattern is consistent with the broader literature on genomic imprinting and quantitative genetics (Ghaouri Kesbi et al., 2025; Wolf et al., 2008).

Spearman’s rank correlations between estimated breeding values obtained from models that included or excluded parental imprinting effects provide insight into how accounting for these effects influences genetic evaluations. A correlation below unity indicates that the ranking of animals may change. In the present study, for traits significantly influenced by imprinting effects (MY1, MY2, FP1, and FP), the correlations between breeding values estimated with and without imprinting effects were high when considering all animals, indicating that the rankings were largely consistent across models. This pattern is consistent with the findings of Perdomo‑Gonzalez et al. (2023) and Ghafouri‑Kesbi et al. (2025).

However, it is also essential to examine Spearman’s rank correlations among the top‑ranked animals, as correlations based on the entire population can mask changes occurring at the extremes (Ghafouri‑Kesbi et al., 2025). Our results showed that animals with high estimated breeding values under the conventional model, without imprinting effects, were also likely to rank highly under the model that included imprinting. Nevertheless, the correlations for the top 1% of animals were noticeably below unity (0.73 for MY1, 0.75 for MY2, 0.81 for FP1, and 0.68 for FP2), indicating that the selection of elite animals may be less accurate when imprinting effects are ignored, particularly under high selection intensity. Therefore, for the Murciano‑Granadina population studied here, a model incorporating paternal imprinting effects for MY1, FP1, and FP2, and both paternal and maternal imprinting effects for MY2, is recommended for genetic evaluation. In this study, imprinting effects for MY1, FP1, and FP2 were of paternal origin, whereas both parental origins were relevant for MY2. Significant paternal imprinting implies that, for imprinted genes, the paternal allele is partially or completely silenced while the maternal allele remains active, or vice versa in the case of maternal imprinting.

### 4.4 Genetic parameters

There is limited published information on genetic parameters for milk yield and composition traits in dairy animals that explicitly accounts for parental imprinting effects. However, in recent years, several studies have examined imprinting influences on growth, fleece weight, and reproductive traits in goats. Mokhtari et al. (2022b) investigated imprinting effects on growth traits and yearling greasy fleece weight in Raeini Cashmere goats. Ehsaninia and Ghafouri‑Kesbi (2024) evaluated maternal imprinting effects on growth and reproductive traits in Murciano‑Granadina goats and reported that maternal imprinting significantly affected kid birth weight and litter size at weaning, traits that should therefore be considered in genetic evaluations. Similarly, Bahreini Behzadi (2026) examined parental imprinting effects on growth and yearling fleece weight in Markhoz goats and emphasized the importance of incorporating imprinting, particularly paternal effects, into the genetic evaluation of body‑weight traits in this breed.

Kaiser et al. (1998) noted that maternal and paternal imprinting effects accounted for 1% and 0.5% of the phenotypic variation in milk yield of Australian Friesian cows, respectively. Essl and Voith (2002) reported maternal‑imprinting heritability estimates of 0.01, 0.01, and 0.00 for milk yield in the first, second, and third lactations of the Austrian Simmental population, along with similarly low or near‑zero values for milk fat and protein percentages across the same lactations. In contrast, Ghafouri‑Kesbi et al. (2025) found higher maternal‑imprinting heritabilities in Iranian Holstein cows, 0.07 ± 0.02 for milk yield, 0.06 ± 0.01 for fat percentage, 0.05 ± 0.01 for protein percentage, and 0.06 ± 0.01 for somatic cell score, and concluded that incorporating imprinting effects improved model fit for all traits examined.

As shown in Table 5, Table 6, the inclusion of parental imprinting effects in the model led to reduced estimates of additive genetic variances. This indicates that omitting imprinting effects inflates additive genetic variance estimates for the traits analyzed in this study. Similar patterns have been reported by Essl and Voith (2002), Meyer and Tier (2012), and Ghafouri‑Kesbi et al. (2025). In the present study, paternal‑imprinting heritability estimates were 0.07, 0.02, 0.07, and 0.10 for MY1, MY2, FP1, and FP2, respectively, while a low maternal‑imprinting heritability of 0.08 was observed for MY2. In contrast to our findings, Ghafouri‑Kesbi et al. (2025) reported that maternal imprinting contributed more substantially to the phenotypic variation of milk yield, fat percentage, protein percentage, and somatic cell score in Iranian Holstein cows.

In the present study, by using the best‑fitting model for each trait, direct heritability estimates fall within the low to moderate range and varied from 0.05±0.02 for FP2 to 0.34±0.05 for PP2. Castaneda‑Bustos et al. (2014) reported higher heritability estimates of 0.37, 0.54, and 0.64 for milk yield, fat percentage, and protein percentage in U.S. dairy goats. Likewise, Mucha et al. (2022), using a meta‑analysis technique, reported heritability estimates of 0.27, 0.48, 0.48, and 0.21 in dairy goats, which were generally higher than those obtained in the present study. Aguirre‑Arroyo et al. (2024) reported heritability estimates for SCS ranging from 0.11 to 0.26 across several dairy goat breeds. Differences in heritability estimates across studies are likely due to variations in population genetic structure, the size and quality of the datasets used, and the statistical models applied.

In the present study, genetic and phenotypic correlations among the same traits across lactations were positive, implying that selection for any of the studied traits in the first lactation period will positively influence the same traits in the second and third lactation periods. All within-period genetic and phenotypic correlations of milk yield with both milk-fat and milk-protein percentages were negative. Therefore, selection for increased milk yield will decrease milk-fat and milk-protein percentages in the first three investigated lactation periods of the studied population of the Murciano-Granadina goat breed. From a physiological standpoint, this pattern is consistent with a dilution effect, in which increased milk yield is driven chiefly by enhanced secretion of lactose and water, whereas fat and protein synthesis fails to increase at the same rate. From a genetic perspective, these correlations may be attributable to pleiotropy or to linkage between loci affecting the traits (Rauw et al., 1998).

On the other hand, within-breed genetic and phenotypic correlations of milk yield and somatic cell score traits were positive, implying that selection for higher milk yield in the first three lactation periods of the studied population of the Murciano-Granadina goat breed would increase somatic cell score and consequently result in higher susceptibility to mastitis. It should be noted that milk somatic cell score is considered an indirect indicator of mastitis in dairy animals (Oliveira et al., 2025). Various explanations have been put forward for the adverse association between milk yield and health in dairy animals. Animals with high yields allocate a substantial portion of their nutrient supply to the mammary gland, yet during early lactation voluntary feed intake is insufficient to satisfy this requirement. The resulting negative energy balance triggers the mobilization of body reserves (Collard et al., 2000), which has been linked to compromised immune function and an elevated risk of metabolic and infectious disorders, such as mastitis (Collard et al., 2000; Rauw et al., 1998). Using a meta-analysis, Mucha et al. (2022) reported genetic correlations between milk yield and somatic cell score, fat percentage and somatic cell score, and protein percentage and somatic cell score in dairy goat breeds of 0.35, −0.19, and −0.06, respectively.

In the present study, the paternal imprinting correlations for MY1-MY2 and FP1-FP2 were positive estimates, while those of MY1-FP1, MY1-FP2, MY2-FP1, and MY2-FP2 were negative ones. Ghaouri-Kesbi et al. (2025) emphasized that the presence of imprinting correlations shows that imprinted genes are not confined to influencing a single trait; instead, they can contribute to variation in multiple traits. At the DNA level, positive imprinting correlations suggest that allelic variants tend to exert effects in the same direction on positively correlated traits when comparing reciprocal heterozygotes (i.e., Aa vs. aA). In practical terms, if the Aa heterozygote exhibits a higher genotypic value than the aA heterozygote for one trait, it is also likely to show a higher value for another positively correlated trait. Conversely, for negatively correlated traits, the sign of this difference would typically be reversed. In the current study, the additive direct genetic correlations share the same sign as the paternal imprinting correlations, which is in agreement with the findings of Ghafouri-Kesbi et al. (2025) in Iranian Holstein dairy cows. This pattern implies that a gene expressed in the classical imprinting manner is expected to contribute similarly through both its additive and imprinting effects to the genetic correlation between traits (Ghafouri-Kesbi et al., 2025).

## 5 Conclusions

This study shows that parental imprinting is a meaningful source of variation for milk‑yield and fat‑percentage traits in the studied population of Murciano‑Granadina goats, with paternal and maternal imprinting jointly affecting MY2. The imprinting effects account for a non‑negligible proportion of phenotypic variance for MY1, MY2, FP1, and FP2, and ignoring them in the models used for genetic analysis of these traits inflates additive variance and distorts selection rankings. These findings support the inclusion of imprinting effects in genetic evaluation models to improve the accuracy of selection and to design more effective breeding programs for these traits. However, further validation in independent herds and populations is required before broader recommendations can be made.

## Declaration of generative AI use

We used Copilot to proofread the manuscript and to correct grammatical and punctuation errors before submission.

## Ethical approval

This study did not involve any experimental procedures on live animals. All data were obtained from routinely collected performance and pedigree records from a commercial goat breeding farm.

## Ethics declaration

This study was conducted in accordance with the following guidelines for animal welfare and/or reporting: This study did not involve any experimental procedures on live animals. All data used were derived from routinely collected performance and pedigree records from a commercial goat breeding farm. Ethics committee approval was not required. This study did not involve any experimental procedures on live animals. All data used were derived from routinely collected performance and pedigree records from a commercial goat breeding farm.

## Funding

This research received no specific funding from public, commercial, or not‑for‑profit agencies.

## CRediT authorship contribution statement

**Morteza Mokhtari:** Writing – original draft, Project administration, Methodology, Formal analysis, Data curation, Conceptualization. **Farhad Ghafouri-Kesbi:** Writing – original draft, Methodology, Investigation, Conceptualization. **Moslem Moghbeli Damane:** Methodology, Data curation, Conceptualization.

## Declaration of competing interest

The authors declare that they have no known competing financial interests or personal relationships that could have appeared to influence the work reported in this paper.

## Data Availability

The data that support the findings of this study are available from the corresponding author upon reasonable request.
