# Supplementary material for: Parental imprinting effects on milk yield and composition traits in Murciano-Granadina goats
Source: Vet Anim Sci. 2026 Oct 26;34:100915. doi: 10.1016/j.vas.2026.100915 (PMC13735291; doi:10.1016/j.vas.2026.100915)
Supplement: Supplementary file 1 [file mmc1.docx]

Supplementary Table 1 Estimates of genetic (r_g_), paternal imprinting (r_pt_), phenotypic (r_p_), and residual (r_e_) correlations among the investigated traits

| Pair traits ^¥^ | r_a_ | r_pt_ | r_p_ | r_e_ |
| --- | --- | --- | --- | --- |
| MY1-MY2 | 0.81±0.10 | 0.68±0.13 | 0.37±0.01 | 0.32±0.02 |
| MY1-MY3 | 0.88±0.23 | - | 0.23±0.02 | 0.18±0.02 |
| MY1-FP1 | -0.63±0.14 | -0.39±0.15 | -0.45±0.04 | -0.38±0.03 |
| MY1-FP2 | -0.38±0.09 | -0.25±0.09 | -0.29±0.02 | -0.19±0.04 |
| MY1-FP3 | -0.25±0.11 | - | -0.16±0.03 | -0.12±0.03 |
| MY1-PP1 | -0.32±0.12 | - | -0.24±0.04 | -0.15±0.03 |
| MY1-PP2 | -0.21±0.07 | - | -0.16±0.02 | -0.10±0.02 |
| MY1-PP3 | -0.18±0.08 | - | -0.09±0.03 | -0.05±0.02 |
| MY1-SCS1 | 0.19±0.06 | - | 0.15±0.04 | 0.12±0.02 |
| MY1-SCS2 | 0.13±0.05 | - | 0.11±0.03 | 0.08±0.03 |
| MY1-SCS3 | 0.09±0.04 | - | 0.10±0.04 | 0.07±0.02 |
| MY2-MY3 | 0.91±0.25 | - | 0.35±0.02 | 0.32±0.02 |
| MY2-FP1 | -0.12±0.05 | -0.08±0.02 | -0.07±0.02 | -0.04±0.03 |
| MY2-FP2 | -0.68±0.12 | -0.23±0.03 | -0.43±0.03 | -0.31±0.02 |
| MY2-FP3 | -0.05±0.04 | - | -0.03±0.02 | -0.02±0.02 |
| MY2-PP1 | -0.17±0.07 | - | -0.09±0.02 | -0.05±0.02 |
| MY2-PP2 | -0.71±0.23 | - | -0.59±0.05 | -0.31±0.03 |
| MY2-PP3 | -0.08±0.08 | - | -0.05±0.03 | -0.02±0.03 |
| MY2-SCS1 | 0.23±0.07 | - | 0.15±0.02 | 0.11±0.01 |
| MY2-SCS2 | 0.36±0.11 | - | 0.18±0.02 | 0.13±0.03 |
| MY2-SCS3 | 0.18±0.07 | - | 0.12±0.04 | 0.09±0.02 |
| MY3-FP1 | -0.07±0.03 | - | -0.04±0.03 | -0.02±0.03 |
| MY3-FP2 | -0.11±0.05 | - | -0.06±0.04 | -0.03±0.02 |
| MY3-FP3 | -0.69±0.21 | - | -0.51±0.06 | -0.45±0.04 |
| MY3-PP1 | -0.12±0.04 | - | -0.05±0.04 | -0.03±0.02 |
| MY3-PP2 | -0.26±0.06 | - | -0.15±0.03 | -0.08±0.02 |
| MY3-PP3 | -0.49±0.11 | - | -0.34±0.02 | -0.25±0.04 |
| MY3-SCS1 | 0.12±0.05 | - | 0.09±0.03 | 0.04±0.02 |
| MY3-SCS2 | 0.26±0.07 | - | 0.17±0.03 | 0.09±0.04 |
| MY3-SCS3 | 0.46±0.13 | - | 0.31±0.02 | 0.25±0.03 |
| FP1-FP2 | 0.15±0.02 | 0.21±0.05 | 0.14±0.03 | 0.18±0.03 |
| FP1-FP3 | 0.11±0.01 | - | 0.16±0.05 | 0.18±0.02 |
| FP1-PP1 | 0.92±0.25 | - | 0.40±0.02 | 0.36±0.03 |
| FP1-PP2 | 0.62±0.12 | - | 0.35±0.03 | 0.23±0.07 |
| FP1-PP3 | 0.48±0.15 | - | 0.41±0.02 | 0.31±0.06 |
| FP1-SCS1 | 0.38±0.18 | - | 0.27±0.03 | 0.26±0.04 |
| FP1-SCS2 | 0.31±0.12 | - | 0.25±0.05 | 0.17±0.05 |
| FP1-SCS3 | 0.28±0.09 | - | 0.19±0.03 | 0.14±0.03 |
| FP2-FP3 | 0.77±0.04 | - | 0.62±0.03 | 0.52±0.04 |
| FP2-PP1 | 0.56±0.16 | - | 0.38±0.06 | 0.26±0.05 |
| FP2-PP2 | 0.75±0.10 | - | 0.48±0.02 | 0.42±0.03 |
| FP2-PP3 | 0.52±0.12 | - | 0.37±0.03 | 0.28±0.03 |
| FP2-SCS1 | 0.31±0.12 | - | 0.19±0.02 | 0.13±0.02 |
| FP2-SCS2 | 0.63±0.13 | - | 0.21±0.02 | 0.12±0.03 |
| FP2-SCS3 | 0.27±0.09 | - | 0.23±0.04 | 0.19±0.04 |
| FP3-PP1 | 0.41±0.13 | - | 0.34±0.06 | 0.28±0.03 |
| FP3-PP2 | 0.38±0.12 | - | 0.28±0.02 | 0.21±0.05 |
| FP3-PP3 | 0.96±0.23 | - | 0.41±0.03 | 0.36±0.04 |
| FP3-SCS1 | 0.25±0.11 | - | 0.16±0.05 | 0.09±0.02 |
| FP3-SCS2 | 0.21±0.08 | - | 0.10±0.04 | 0.06±0.02 |
| FP3-SCS3 | 0.92±0.26 | - | 0.10±0.03 | 0.53±0.03 |
| PP1-PP2 | 0.54±0.05 | - | 0.36±0.04 | 0.29±0.06 |
| PP1-PP3 | 0.41±0.05 | - | 0.32±0.10 | 0.21±0.02 |
| PP1-SCS1 | 0.71±0.28 | - | 0.46±0.02 | 0.44±0.03 |
| PP1-SCS2 | 0.23±0.09 | - | 0.19±0.02 | 0.12±0.02 |
| PP1-SCS3 | 0.18±0.08 | - | 0.13±0.03 | 0.10±0.03 |
| PP2-PP3 | 0.43±0.04 | - | 0.38±0.03 | 0.35±0.06 |
| PP2-SCS1 | 0.17±0.07 | - | 0.12±0.04 | 0.09±0.02 |
| PP2-SCS2 | 0.88±0.07 | - | 0.34±0.02 | 0.15±0.04 |
| PP2-SCS3 | 0.25±0.06 | - | 0.15±0.03 | 0.11±0.02 |
| PP3-SCS1 | 0.12±0.04 | - | 0.18±0.04 | 0.10±0.03 |
| PP3-SCS2 | 0.15±0.06 | - | 0.08±0.02 | 0.06±0.03 |
| PP3-SCS3 | 0.94±0.21 | - | 0.16±0.03 | 0.45±0.06 |
| SCS1-SCS2 | 0.23±0.03 | - | 0.14±0.02 | 0.12±0.04 |
| SCS1-SCS3 | 0.26±0.04 | - | 0.11±0.03 | 0.13±0.04 |
| SCS2-SCS3 | 0.24±0.03 | - | 0.11±0.03 | 0.13±0.05 |

^¥^ MYi: total milk yield in the i^th^ lactation period, FPi: average fat percent in the i^th^ lactation period, PPi: average protein percent in the i^th^ lactation period, SCSi: average somatic cell score in the i^th^ lactation period. i: 1, 2, and 3, which stand for the first, second, and third lactation period, respectively.
